# Roles of Psychiatry, Dietary, and Oncology Services in Occupational Medicine: A Comprehensive Review

**DOI:** 10.7759/cureus.98069

**Published:** 2025-11-29

**Authors:** Joshua A Jogie, Zahir Mohammed, Tricia Bobb, Zara Suite-Stewart, Shivan Ali

**Affiliations:** 1 Occupational Health Unit, St. James Medical Complex, Port of Spain, TTO; 2 Oncology Department, St. James Medical Complex, Port of Spain, TTO; 3 Psychiatry Department, Arima General Hospital, Arima, TTO; 4 Nutrition and Dietetics Department, St. James Medical Complex, Port of Spain, TTO

**Keywords:** dietary services, occupational medicine, oncology, psychiatry, return-to-work

## Abstract

Occupational medicine manages the interface between health and work. Many employees live with mental illness, cancer, or diet-related diseases in safety-critical roles. These burdens change attention, stamina, risk, and retention. Psychiatry stabilizes mood, sleep, cognition, and substance use. Dietetic services improve energy balance, hydration, and micronutrients. Oncology aligns staging, treatment, and survivorship with duty risk. Services are often siloed, delaying care and widening inequity. Teams need clear roles, shared tools, and models that fit shifts, heat, chemicals, and job design. This article aims to synthesize the roles of psychiatric, dietetic, and oncology services in occupational medicine; to describe feasible models across prevention, clinical management, assessment, and return to work; and to propose simple tools and metrics for routine use. We searched peer-reviewed studies, guidelines, and consensus statements about adult workers. We included interventions usable in routine practice, such as brief screens, counseling, task modification, medication management, and coordinated care. Findings were mapped to prevention, clinical management, work capacity assessment, and sustained return to work. We extracted setting, workforce, elements, and outcomes, including incidents, sickness absence, presenteeism, claims, and retention. Strength of evidence was summarized pragmatically. In psychiatry, depression, anxiety, PTSD, insomnia, ADHD, and substance use are common. Brief case finding supports safe staffing. CBT, trauma-informed support, motivational interviewing, and monitored medication reduce incidents and days lost. Symptoms should be translated into task limits; for example, avoid lone work during acute suicidality and night duty while insomnia persists. In dietetic services, nutritional risk drives cardiometabolic disease, anemia, dehydration, heat illness, and fatigue; shift work disrupts glycemic control. Actions include brief screens, energy and hydration plans for heat, and alignment of diabetes meals and medication to prevent hypoglycemia. Healthy canteens, cool water, and breaks lower fatigue and errors. In oncology, duties should be aligned with staging, treatment intent, and expected adverse effects, including those affecting infection risk, cytopenias, neuropathy, and lymphedema. During treatment, immunosuppression, anemia, and nausea restrict tasks; measures include vaccine review, substitution away from biohazards and heavy lifting, and compression for lymphedema. Shared tools improve coordination: a joint problem list linking symptoms to tasks; a one-page plan with limits, controls, and review dates; and simple metrics such as incidents, near misses, absence, work ability, and worker-reported outcomes. Implementation improves when confidentiality is protected, stigma is reduced, supervisors are trained, and reviews are fair. Integrated psychiatric, dietetic, and oncology input can make work safer and more inclusive. Begin with a joint task-risk review, match brief evidence-based actions to the job, and set clear fitness and re-evaluation points. Employers should provide healthy food, cool water, fatigue controls, and time for care. Clinics should use validated screens, simple nutrition checks, and work ability indices and record accommodations plainly. Research should test pragmatic bundles and report outcomes that matter to workers and supervisors, including incidents, stable return to work, and equity.

## Introduction and background

Occupational medicine aims to keep workers safe, healthy, and productive. Many workers now live longer with chronic conditions. They also face fast-paced work, rotating shifts, and new hazards. This mix changes the role of clinical services that support work.

Psychiatry, dietary, and oncology services each add tools for prevention, assessment, and return to work. They were chosen here because they are common specialist touchpoints for working adults, have strong links to work ability and safety, and illustrate cross-cutting themes: fatigue, cognition, metabolic risk, infection risk, and functional reserve. They are used as exemplars of how specialist input can align with occupational decisions, not as an exhaustive list of all relevant services.

This review sets out why those roles matter in modern workplaces. It explains how these services can align with risk assessment, surveillance, and accommodations. It also identifies gaps where integrated care can improve outcomes, especially the lack of simple, work-focused models that connect specialist services and occupational teams through shared tools and thresholds.

Work affects mental health, and mental health affects work. Global guidance recommends actions at organizational, managerial, and individual levels to protect mental health, support people living with mental illness, and guide return to work. These recommendations cover workload, autonomy, civility, sleep, training, and access to care. They also call for structured plans during the return-to-work period. Occupational clinicians can use this guidance to shape policies, design screening pathways, and time referrals. Clear governance, confidentiality, and worker choice need to anchor any program [1,2].

Evidence links work stressors such as low support, high demands, job insecurity, long hours, and moral injury to higher risks of depression, anxiety, and burnout. Work-related stress is common. Reviews show consistent associations with depressive symptoms across sectors. Screening programs appear acceptable, but do not help unless they trigger effective support and changes at work. Health systems should avoid stand-alone “identify and refer” models that do not adjust working conditions. Fit-for-duty decisions should weigh symptom severity, safety-critical tasks, medication side effects, and relapse risk. Plans should also consider stigma, job control, and supervisor behavior. This needs joint work between the occupational team and psychiatry, with explicit goals tied to function and safety [3-6].

Psychiatric care that targets work outcomes shows benefits. Work-focused cognitive behavioral therapy improves return-to-work metrics for common mental disorders. Collaborative care for depression in primary care settings improves both symptoms and productivity. Insomnia is a key modifiable risk in many jobs. Cognitive behavioral therapy for insomnia improves sleep and reduces daytime impairment. Better sleep links to fewer errors and accidents, especially in safety-critical roles. Occupational teams can embed brief cognitive behavioral therapy for insomnia (CBT-I), sleep hygiene, and shift-scheduling advice in routine programs. When medication is needed, prescribers should consider sedation, reaction time, and role demands. They should time dosing to minimize on-shift effects. Fitness notes should state specific restrictions (e.g., “no night driving” or “no high-risk ladder work”) rather than generic “light duties.” This keeps plans concrete and auditable [7-11].

Some workers need specialized psychiatric input. Post-traumatic stress disorder can follow severe workplace events. Early trauma-focused care and careful, graded exposure at work can help. Substance and sedative use also affect safety. Benzodiazepines increase occupational injury risk. Policies should promote non-sedating treatments for anxiety and sleep problems in safety-sensitive roles. Adult attention-deficit/hyperactivity disorder impairs sustained attention and working memory. Treatment plus environmental supports, including reduced interruptions, checklists, and time-boxing, can reduce errors and support accommodations. Occupational mental health should prioritize simple, measurable targets and frequent follow-up during early work re-entry [12-16].

Dietary services play several roles in occupational medicine. First, they address shift-related metabolic risks. Epidemiology links night and rotating shifts with a higher incidence of type 2 diabetes. Risk appears to rise with duration of night work and may be stronger in some groups. This supports targeted prevention for shift workers: meal timing aligned with circadian biology, caloric distribution earlier in the awake period, and reduced overnight energy intake. Dietary services can deliver clear plans that match roster patterns. They can also coordinate with scheduling to protect consistent eating windows on long blocks of nights [17-19].

Second, dietitians can run workplace interventions that improve food choices and, in some cases, metabolic markers. Cafeteria labeling and point-of-purchase nudges increase healthy purchasing over the years, even if weight effects are small. Worksite diabetes prevention programs adapted from national curricula can improve diet quality and activity. For shift workers, trials suggest that meal timing and lower-energy night meals are feasible and acceptable, with emerging signals for better glucose control. Occupational services should set performance metrics tied to purchasing data, hemoglobin A1c in at-risk groups, and participation rates. Procurement and catering contracts should name these metrics up front [20-25].

Third, dietitians contribute to heat-stress prevention. Heat exposure impairs cognition, strength, and endurance. It raises injury risk and can cause acute kidney injury in certain settings. Structured “rest-shade-hydration” programs and task redesign improve productivity and reduce heat illness. Randomized trials in hot environments show that targeted cooling, shaded rest breaks, and hydration planning reduce symptoms and maintain output. Occupational teams should pair wet-bulb globe temperature monitoring with hydration targets, electrolyte access, and micro-break scheduling. Dietitians can tailor sodium and fluid plans to the task, climate, and worker health status. They can also support acclimatization and recovery nutrition during heat waves [26-28].

Oncology services have a growing influence on occupational decisions. Many workers return to work during or after cancer therapy. Return to work benefits income, identity, and well-being. However, barriers include fatigue, neuropathy, lymphedema, cognitive changes, and fear of relapse. Reviews show diverse needs by cancer type, treatment, job demands, and contract type. A structured, worker-centered process helps early discussion of work goals, risk mapping of core tasks, and staged duty modification. Oncology, rehabilitation, and occupational teams should agree on a simple function-based plan that states what the worker can do now, what will change over time, and what triggers a review [29].

Chemotherapy-induced peripheral neuropathy affects sensation, balance, and dexterity. It raises fall risk and can limit fine motor work or work at heights. Network meta-analysis and reviews show persistent symptoms in many survivors. The occupational exam should include numbness mapping, vibration sense, two-point discrimination for hand tasks, Romberg and tandem stance, and grip strength. Duty restrictions should address ladder use, unprotected elevated work, hot surfaces, and high-precision repetitive tasks. Physical therapy and protective equipment (e.g., anti-slip footwear, padded gloves for vibration) may be needed. Duty progression should follow functional gains, not dates on the calendar [30-33].

Lymphedema affects endurance and handling tasks. It can limit heavy lifting and sustained overhead work and cause time loss for compression and self-care. Studies show work disruption and career impact, yet progressive resistance training is generally safe under supervision. Occupational plans can allow lifting within symptom-limited ranges with compression garments and close monitoring. Employers should offer ergonomic changes, lift aids, and task rotation. Early referral to oncology rehabilitation and lymphedema therapy reduces flare frequency and improves function [34-38].

Infection risk matters for workers on cytotoxic chemotherapy or biologics. Neutropenia increases the risk from crowded or poorly ventilated spaces and from tasks with frequent contact with sick persons. For low-risk febrile neutropenia, ambulatory care is possible with strict criteria and rapid access. In the workplace, planning should include flexible leave for urgent evaluation, an exposure response plan, and temporary reassignment away from high-exposure tasks during periods of low counts. Vaccination status and timing should be reviewed with oncology teams. The fit note should specify objective criteria (e.g., “avoid direct high-exposure contact tasks until absolute neutrophil count (ANC) recovers above a defined threshold”) rather than vague “avoid sick contacts.” Clear criteria simplify supervisor action and reduce conflict [39].

Fatigue is a common, cross-cutting issue in oncology, depression, and shift work. It reduces vigilance and increases error risk. Occupational teams should use brief tools to track work capacity and productivity alongside symptoms. The Work Ability Index correlates with mental health and predicts absence in healthcare workers and other groups. The Work Productivity and Activity Impairment questionnaire captures presenteeism and absenteeism in trials and practice. Using both gives a picture of capacity at work and time away from work. Results can trigger the right referral: psychiatry when mood or sleep drive fatigue; dietetics when energy intake, hydration, or glycemia play a role; and oncology rehab when deconditioning or treatment effects dominate [40,41].

Integration depends on timing. For new mental health symptoms, early psychiatric input reduces delay, prevents risk escalation, and supports job retention. For shift workers, dietetic input has the best value before roster changes or during onboarding to nights. For cancer care, oncologists should discuss work at diagnosis and revisit it before each major treatment phase. Shared digital notes using function-based language help coordination: “can stand 30 minutes at a time,” “no night driving,” “needs predictable 30-minute meal breaks between 22:00 and 02:00,” and “avoid ladders until vibration sense improves.” These statements translate into tasks and schedules. They also make it easier to audit accommodations.

Patient privacy and consent are critical. Workers need to know what will be shared. Fit notes should omit diagnoses unless the worker chooses to disclose them. They should focus on restrictions, capacities, and duration. Supervisors need training to read and apply these notes, avoid stigma, and escalate concerns to occupational health rather than to peers. Unions or worker councils can help with policy design and trust. Short, frequent check-ins align with the fluctuating course of mental health and cancer symptoms.

Equity matters. People in precarious jobs, shift work, or physically demanding roles often have less control and less access to clinical services. They also face higher risks of diabetes, heat stress, and injury. Occupational medicine should deliver services where risk is highest. Examples include dietitian-led meal timing clinics for night workers, portable shade and hydration kits for outdoor crews, and on-site CBT-I groups for rotating shift teams. Oncology rehab should offer evening or weekend sessions. These steps improve uptake and fairness.

Safety must guide fitness and return to work. In safety-critical roles, small cognitive or sensory changes can have large effects. Occupational teams should consider simulation or practical skills checks before lifting restrictions. For example, a utilities worker with neuropathy may need a supervised climb-and-balance evaluation. A professional driver with insomnia may need an on-road assessment after CBT-I and before resuming long night routes. Shared thresholds, such as accident-free observation periods or validated balance scores, can support these decisions. For some combinations of condition and task, such as acute suicidality or unstable severe symptoms in safety-critical roles, temporary removal from those duties is required regardless of specialist input. Integration then helps plan a safe, staged return when risk is controlled.

Finally, outcomes need to be measured. Programs should track participation, symptom change, work ability, injury rates, and productivity. They should also track process metrics: time to first specialist visit, time to accommodations, and worker satisfaction. Data should drive improvement. If screening finds high needs but uptake is low, fix access barriers. If cafeteria nudges shift choices but weight does not change, add targeted counseling for at-risk groups. If return-to-work plans stall, revisit job redesign or graded exposure steps.

Psychiatry stabilizes mood, sleep, cognition, and behavior; dietary services support metabolic health, circadian alignment, and heat resilience; and oncology services manage function, risks, and phased return after cancer. When these services integrate with occupational risk management, workers stay healthier and safer, and organizations retain skills.

Methods

This is a narrative review. We searched peer-reviewed studies, guidelines, and consensus statements focused on adult workers and work-relevant outcomes. Structured searches were performed in PubMed and major guideline repositories from 2000 onwards, using terms related to mental health, diet and nutrition, shift work, heat stress, cancer, return to work, and occupational health. Reference lists of key articles and guidelines were also screened.

We prioritized systematic reviews, meta-analyses, randomized and quasi-experimental trials, and major guidelines with clear implications for work function, safety, or return to work. We included interventions usable in routine practice, such as brief screens, counseling, task modification, medication management, and coordinated care. We excluded pediatric studies and reports without any health, functional, or work-related outcomes. We did not conduct a formal risk-of-bias assessment or meta-analysis. Evidence is summarized pragmatically by theme, with emphasis on occupational relevance and feasibility.

## Review

Cross-cutting occupational themes

Occupational medicine works best when clinical services align with work demands. Three services are central in this review. Psychiatry stabilizes mental health and protects safety. Dietary services reduce cardiometabolic risk and support performance. Oncology services coordinate safe work for people with cancer.

Across these domains, several themes recur: fatigue, cognition, physiological reserve, infection risk, and equity. The focus is simple: screen early, act on modifiable risks, match duty to capacity, and measure work functioning in real time. We emphasize interventions that change what people do at work, not only what they feel. We also center measurement. Without structured measures of work role functioning and productivity loss, programs drift. With them, teams can set targets and adjust care plans.

Many mild-to-moderate problems, such as common anxiety, simple insomnia, and basic nutritional issues, can be managed by occupational physicians, primary care teams, and psychologists. Specialist psychiatric, dietetic, or oncology input is most useful for complex, persistent, or safety-critical presentations, or when treatment side effects have clear implications for duty.

Mental health and psychiatric input

Psychiatry in the workplace is not a luxury. It is an essential control for common risks: depression, anxiety, trauma, insomnia, attention-deficit/hyperactivity disorder (ADHD), and substance use. Collaborative models improve symptoms and work functioning. In a randomized trial among employed adults, telephone-based depression care management increased treatment initiation and improved work productivity compared with usual care [42]. Work-focused psychotherapy is more effective for return to work than symptom-focused therapy alone. A trial that combined work-focused cognitive behavioral therapy with individual job support shortened sickness absence and improved mental health outcomes [43]. These programs use brief, scheduled contacts, explicit work goals, graded exposure to workplace tasks, and direct communication with supervisors about modified duty.

Insomnia is a core driver of presenteeism and injury. It is also highly treatable with CBT-I. A placebo-controlled randomized trial of fully automated online CBT-I reduced insomnia severity and improved daytime functioning, showing a scalable path for employer health programs and for clinicians who manage shift workers [44]. Fatigue risk management systems complement clinical care. They set clear limits on consecutive night shifts, prescribe rest opportunities, and train supervisors to monitor alertness. A foundational review shows that structured fatigue systems reduce risk when paired with evidence-based sleep interventions [45]. Medication choices also affect safety-critical tasks. Observational data associate sedative-hypnotic use with an increased risk of motor vehicle crashes, which has immediate implications for workers who drive or operate equipment [46]. When hypnotics are needed, clinicians should select agents with shorter half-lives, restrict use to the lowest effective dose, avoid co-administration with alcohol or other central nervous system (CNS) depressants, and pair them with CBT-I. For serious mental illnesses, evidence supports individual placement and support (IPS) to achieve competitive employment; a recent meta-analysis confirms superior job outcomes versus traditional approaches [47]. IPS should sit alongside psychiatric care within occupational programs for appropriate workers.

Measurement anchors the psychiatric role. The Work Limitations Questionnaire (WLQ) estimates on-the-job productivity loss across time, physical, mental, and output demands and shows strong reliability and validity for workplace use [48]. The Work Role Functioning Questionnaire 2.0 (WRFQ 2.0) captures the capacity to meet work role demands and has been validated generally [49] and in cancer survivors [50]. These tools complement symptom scales and reveal what matters to employers and workers: consistent task performance, schedule adherence, safety behavior, and output. Results should feed into duty plans. A drop in scores for mental demands or output justifies temporary accommodations, closer follow-up, or a switch to work-focused therapy.

Metabolic, circadian, and nutritional considerations

Dietary services address two problems that erode work capacity: poor food environments at work and circadian misalignment from shift work. Circadian misalignment raises glucose, blood pressure, and cortisol and reduces sleep efficiency even in healthy adults [51]. In real-world night work, circadian disruption also changes energy expenditure and eating patterns in ways that hinder weight control [52]. Dietitians can reduce this burden with environmental and timing interventions.

Worksite “traffic-light” labeling and choice architecture in a large hospital system sustained reductions in calories purchased over two years, driven by a shift away from the least healthy items [53]. Calorie labeling alone shows mixed effects, with one stepped-wedge pilot indicating feasibility but variable impact across sites; operational issues matter, and design details determine success [54]. The signal is clear: interventions that change defaults and placement outperform passive education.

Meal timing during night work is a modifiable lever. A randomized crossover study in night-shift police officers found that fasting during the night shift altered next-day energy intake patterns and lowered morning insulin and insulin resistance metrics, showing metabolic effects of timing independent of total calories [55]. A later cluster-randomized trial in simulated night-shift work reported that fasting attenuated the adverse effects of circadian misalignment on glucose metabolism, suggesting a practical protocol for select workers under clinical supervision [56]. These timing strategies should be paired with simple rules: schedule the main caloric load on day shifts, emphasize protein and fiber early in the shift, avoid large, late-night carbohydrate loads, and maintain hydration.

Heat is a dietary and hydration problem as much as an environmental one. In hot field conditions, rest-shade-hydration programs improve kidney health and can preserve or increase productivity even when they shorten nominal work time [57]. Laboratory simulations show that hydration, shade, and body cooling lower core and skin temperatures and support physical work output without harm, reinforcing a layered mitigation plan for high-heat tasks [58]. Occupational clinicians should standardize hydration plans by task and heat index, ensure cool rest areas, and train supervisors to recognize early heat illness, particularly in settings where chronic kidney disease of nontraditional origin is a risk.

Cancer survivorship and oncology-related work issues

Oncology services in occupational medicine aim to maintain safe employment during treatment and speed return to work after treatment while protecting health. This requires risk stratification, infection control, neurotoxicity management, rehabilitation, and employer coordination. For infection risk, ambulatory care is reasonable for carefully selected patients with febrile neutropenia. The Multinational Association of Supportive Care in Cancer (MASCC) risk index remains a practical tool; a score ≥21 identifies adults at lower risk for serious complications [59], and multicenter validation supports its use [60]. More recent evidence synthesizes performance against other tools, such as the Clinical Index of Stable Febrile Neutropenia (CISNE), and supports accuracy for predicting complications when applied appropriately [61]. In practice, clinicians should integrate MASCC scoring with a workplace exposure review: public-facing roles, shared vehicles, poorly ventilated spaces, and close-contact tasks may justify temporary reassignment or remote work during high-risk periods.

Chemotherapy-induced peripheral neuropathy impairs fine motor control, gait, and endurance, which can increase fall risk and reduce the capacity for vibration, forceful gripping, and repetitive hand tasks. Updated recommendations emphasize prevention when possible, patient education, medication review, and functional assessment; routine dosing of supplements lacks strong support, and exercise and symptom monitoring are key [62]. Broader reviews also outline mechanisms and realistic expectations for symptom control rather than cure [63]. Occupational clinicians should translate these points into job analyses: avoid vibrating tools, set limits on repetitive force, add anti-slip footwear and handrails, and use sit-stand options to reduce endurance demands. For safety-critical roles, functional testing should precede return to duty.

Return-to-work processes benefit from early, coordinated, and work-focused support embedded in oncology care. A systematic review showed that interventions integrated into cancer care, not bolted on later, had more favorable work outcomes [64]. A randomized trial of early, tailored work-related support during cancer treatment improved work readiness, underscoring the value of proactive planning and individualized accommodations [65]. Work role measures help here, too. The WRFQ 2.0, validated in cancer survivors, detects persistent limitations across scheduling, output, and physical and mental demands and can guide graded return plans [50]. Duty plans should be explicit: start with shortened shifts or reduced task variety, limit lifting and repetitive hand tasks for neuropathy, control exposure risk during expected nadirs, and set objective step-ups tied to symptom and function thresholds. Clear criteria avoid conflict and reduce failed returns.

Some scenarios in active cancer care and mental health are intrinsically incompatible with specific safety-critical tasks. In those cases, specialist input cannot replace temporary removal from those tasks. Its role is to clarify risk, define conditions for safe re-exposure, and help design a phased and realistic return plan.

Integration model

Integration across psychiatry, dietary, and oncology services matters because workers present with overlapping problems. A worker on adjuvant chemotherapy may also have insomnia and anxiety; a night-shift nurse may struggle with weight, fatigue, and mood. Care should be coordinated through a single work-focused plan.

Here is a simple sequence. First, define the job’s critical tasks and risks. Second, measure work role functioning with WLQ or WRFQ and baseline symptom scales. Third, agree on near-term goals that match capacity and safety needs. Fourth, deploy high-yield interventions: CBT-I instead of long-term hypnotics [44-46], work-focused CBT with job support for stress-related absence [43], IPS when severe mental illness blocks competitive employment [47], cafeteria labeling coupled with product placement [53,54], and night-shift meal-timing protocols for those with metabolic risk [55,56]. Fifth, set a monitoring loop using objective work metrics (attendance, errors, near misses, output) plus repeated WLQ/WRFQ scores [48-50]. Sixth, tighten or relax accommodations based on data. For oncology cases, layer in neutropenia risk management and neuropathy-informed task limits [59-63] and start return-to-work planning during treatment, not after [64,65].

This approach reduces two common errors. One is treating symptoms in isolation from job demands. The other is setting vague return targets. Both raise costs and frustrate workers and supervisors. Measurable work outcomes keep everyone aligned. The psychiatric role can set CBT agendas around graded exposure to the specific tasks that workers fear or avoid. The dietitian can redesign the actual food choices at the worksite and adjust timing to the shift plan. The oncology clinician can translate regimen risks into concrete duty limits and timelines. Supervisors need simple rules: remove barriers, control exposures, and check progress against agreed metrics. When services share the same targets, handoffs improve. Examples of duty restrictions (e.g., ladder work or night driving) are illustrative and must be adapted to the industrial, legal, and cultural context.

Practical tools, metrics, and implementation

Several cross-cutting implementation points deserve emphasis. First, digital tools make access cheaper and faster. Online CBT-I has strong evidence and fits shift schedules [44]. Digital self-monitoring of sleep, meals, and symptoms supports coaching but should be used to inform, not police. Second, medication safety must be job-specific. Sedating agents that impair driving or vigilance require active risk management and time-limited prescribing [46]. Third, environment beats education. For diet, changing defaults works better than lectures [53,54]. For heat, rest-shade-hydration with cooling outperforms advice alone [57,58]. Fourth, timing matters. For night shift, aligning meals and sleep with circadian biology reduces metabolic strain [51,55,56]. Fifth, equity matters. Lower-wage workers often face the highest heat, shift, and infection risks. Interventions should be designed for these settings first, not last.

Finally, programs should plan for evaluation. Choose two primary outcomes (e.g., sustained return to work and incident injury) and one cost metric. Add a small battery of shared process measures across services: WLQ or WRFQ for work role functioning [48-50]; average daily calories purchased from the worksite over time [53,54]; and adherence to graded return plans for oncology [64,65]. Additionally, build escalation rules (e.g., if insomnia persists after four weeks of CBT-I, add pharmacotherapy with a safety review; if WRFQ-mental demands scores remain low after work-focused CBT, consult IPS [43,47]), and keep plans flexible. Work is not static, and neither is health.

Limitations and contextualization

Regulation, case law, and resources differ across jurisdictions. Fit-for-duty, confidentiality, and return-to-work decisions must follow local statutes, regulatory guidance, and collective agreements. This review offers general clinical and organizational principles rather than legal advice. Implementation will depend on local service capacity and workforce mix. In many settings, occupational physicians, general practitioners, nurses, and psychologists will deliver much of the care described here, with specialist psychiatry, dietetics, and oncology input reserved for selected cases.

## Conclusions

Integrated psychiatric, dietary, and oncology input helps people work safely and stay employed. Use work-focused psychotherapy, IPS when needed, CBT-I, fatigue risk management systems, and cautious prescribing; redesign food choices at work and adjust meal timing for shift teams; and standardize rest-shade-hydration and cooling in heat. In cancer care, plan work early, use function-based restrictions, manage neutropenia risk with clear criteria, and address neuropathy with task limits and rehabilitation. Measure what matters: work role functioning, incidents, absence, and output; set thresholds to step duties up or down; protect privacy, train supervisors, and write one-page plans with explicit restrictions, controls, and review dates; aim for simple changes that alter tasks and environments, not education alone; and start small, test, and scale what works. Employers and clinics should fund these integrated pathways and share results. Researchers should trial pragmatic bundles with worker-centered outcomes. This approach keeps work safe and feasible during mental illness, cancer treatment, and metabolic risk.
